# Supplementary material for: Structure and mechanism of the Nap adhesion complex from the human pathogen Mycoplasma genitalium
Source: Nat Commun. 2020 Jun 8;11:2877. doi: 10.1038/s41467-020-16511-2 (PMC7280502; doi:10.1038/s41467-020-16511-2)
Supplement: Supplementary file 1 — Supplementary Information [file 41467_2020_16511_MOESM1_ESM.pdf]

Supplementary Information

Structure and mechanism of the Nap adhesion complex from the human pathogen *Mycoplasma genitalium*

David Aparicio, Margot P. Scheffer, Marina Marcos-Silva et al.

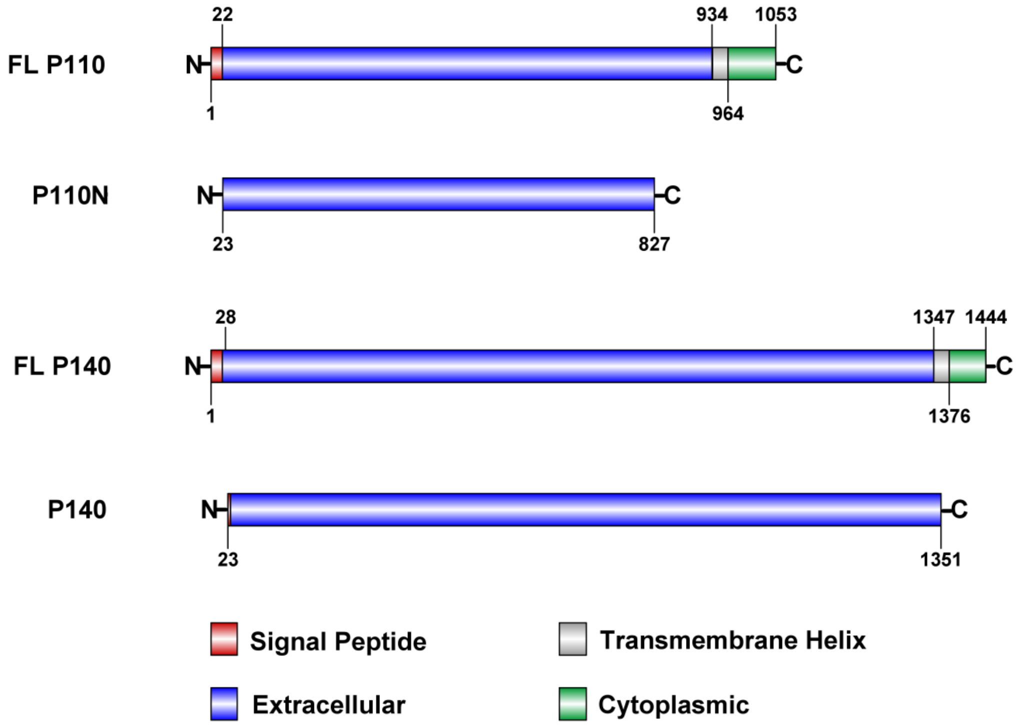

Supplementary Figure 1. **Proteins and constructs used in this study**

The Psi-Pred server[1] was used to predict the location of signal peptides (red), extracellular regions (lilac), transmembrane helices (grey) and cytoplasmic regions (green) in the full length (FL) P140 and P110 proteins. Constructs of the extracellular regions of P140 and P110 are also shown. In P110N the C-domain was excluded. Numbers indicate the residues at the boundaries.

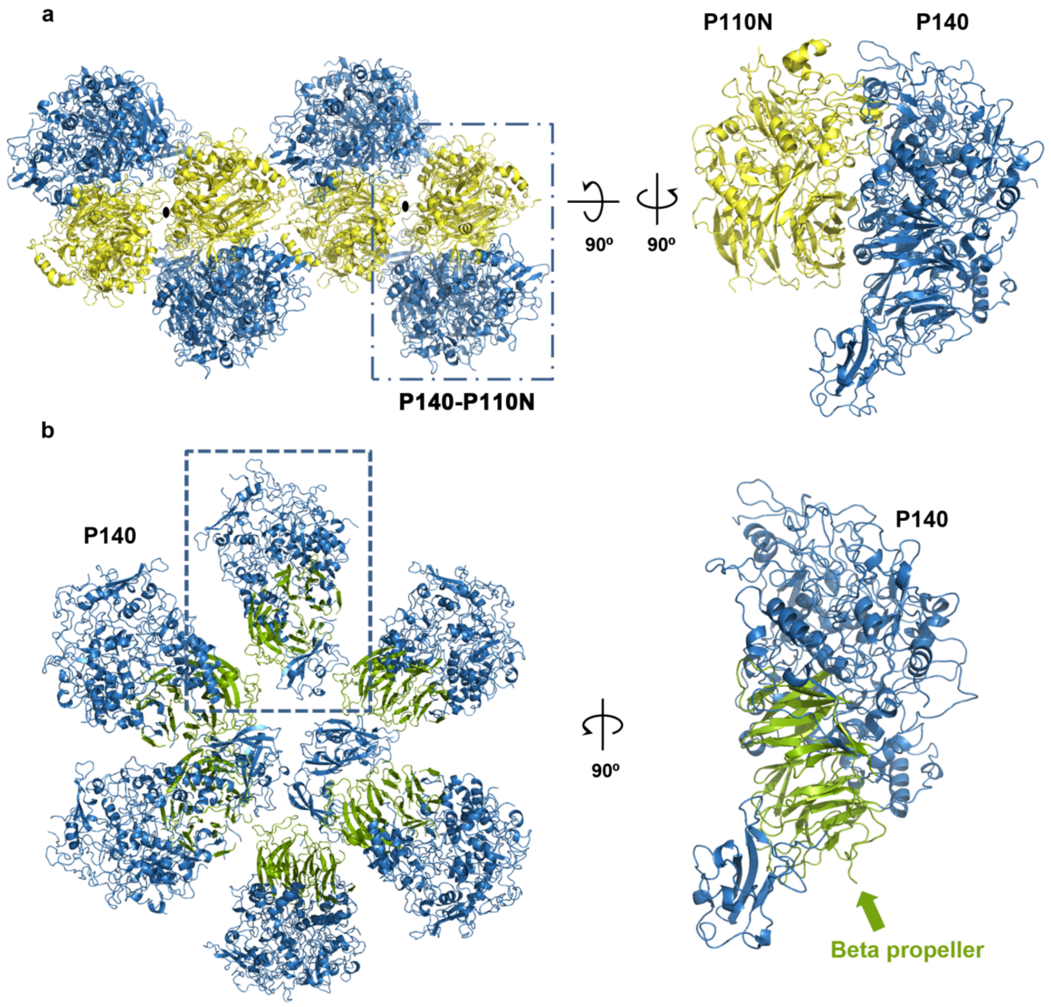

Supplementary Figure 2. **Asymmetric units content of P140-alone and P140-P110N crystals**

a) The P140-P110N crystals contain four P140-P110N heterodimers in the asymmetric unit that are arranged as two dimers with a local, non-crystallographic, two-fold axis (black oblong dots). The P140 construct (blue) includes the N- and C-domains, while the P110N construct (yellow) corresponds only to the N-domain of P110. b) The P140-alone crystals contain six P140 subunits in the asymmetric unit. β-propellers are explicitly indicated (green).

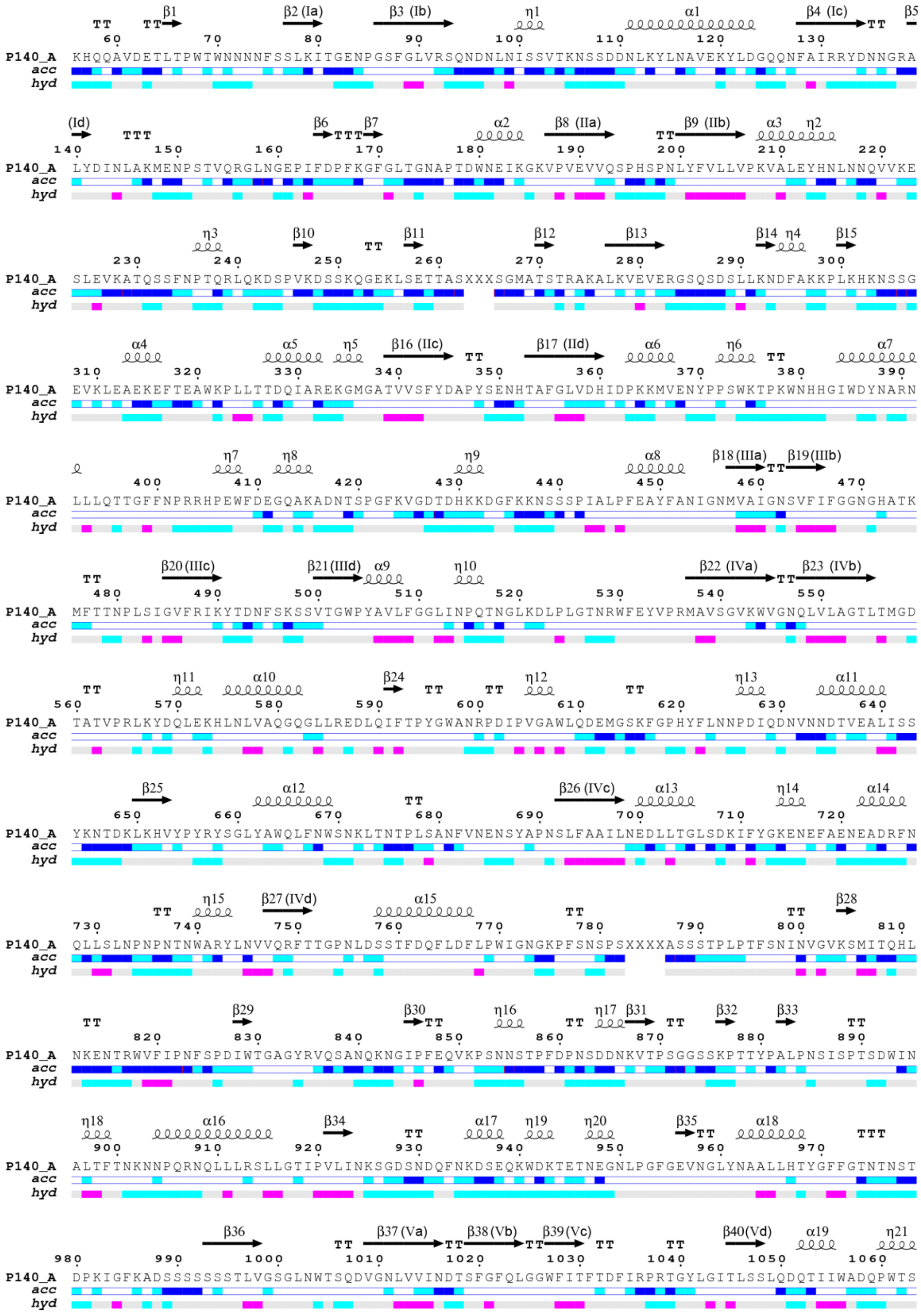

TT TT β1 β2 (Ia) β3 (Ib) η1 α1 β4 (Ic) TT β5
60 70 80 90 100 110 120 130
P140_A KHQQAVDETLTPWTWNNNNFSSLKITGENPGSFGLVRSQNDNLNISSVTKNSSDDNLKYLNAVEKYLDGQQNFAIRRYDNNGRA
acc
hyd
(Id) TTT β6 TTT β7 α2 β8 (IIa) TT β9 (IIb) α3 η2
140 150 160 170 180 190 200 210 220
P140_A LYDINLAKMENPSTVQRGLNGEPIFDPFKGFGLTGNAPTDWNEIKGKVPVEVVQSPHSPNLYFVLLVPKVALEYHNLNNQVVKE
acc
hyd
η3 β10 TT β11 β12 β13 β14 η4 β15
230 240 250 260 270 280 290 300
P140_A SLEVKATQSSFNPTQRLQKDSPVKDSSKQGEKLSETTASXXXSGMATSTRAKALKVEVERGSQSDSLLKNDFAKKPLKHKNSSG
acc
hyd
α4 α5 η5 β16 (IIc) TT β17 (IId) α6 η6 TT α7
310 320 330 340 350 360 370 380 390
P140_A EVKLEAEKEFTEAWKPLLTTDQIAREKGMGATVVSFYDAPYSENHTAFGLVDHIDPKKMVENYPPSWKTPKWNHHGIWDYNARN
acc
hyd
η7 η8 η9 α8 β18 (IIIa) TT β19 (IIIb)
400 410 420 430 440 450 460 470
P140_A LLLQTTGFFNPRRHPEWFDEGQAKADNTSPGFKVGDTDHKKDGFKKNSSSPIALPFEAYFANIGNMVAIGNSVFIFGGNGHATK
acc
hyd
TT β20 (IIIc) β21 (IIId) α9 η10 β22 (IVa) TT β23 (IVb)
480 490 500 510 520 530 540 550
P140_A MFTTNPLSIGVFRIKYTDNFSKSSVTGWPYAVLFGGLINPQTNGLKDLPLGTNRWFEYVPRMAVSGVKWVGNQLVLAGTLTMGD
acc
hyd
TT η11 α10 β24 TT TT η12 TT η13 α11
560 570 580 590 600 610 620 630 640
P140_A TATVPRLKYDQLEKHLNLVAQGQGLLREDLQIFTPYGWANRPDIPVGAWLQDEMGSKFGPHYFLNNPDIQDNVNNDTVEALISS
acc
hyd
β25 α12 TT β26 (IVc) α13 η14 α14
650 660 670 680 690 700 710 720
P140_A YKNTDKLKHVYPYRYSGLYAWQLFNWSNKLTNTPLSANFVNENSYAPNSLFAAILNEDLLTGLSDKIFYGKENEFAENEADRFN
acc
hyd
TT η15 β27 (IVd) α15 TT TT β28
730 740 750 760 770 780 790 800 810
P140_A QLLSLNPNPNTNWARYLNVVQRFTTGPNLDSSTFDQFLDFLPWIGNGKPFSNSPSXXXXASSSTPLPTFSNINVGVKSMITQHL
acc
hyd
TT β29 β30 TT η16 TT η17 β31 TT β32 β33 TT
820 830 840 850 860 870 880 890
P140_A NKENTRWVFIPNFSPDIWTGAGYRVQSANQKNGIPFEQVKPSNNSTPFDPNSDDNKVTPSGGSSKPTTYPALPNSISPTSDWIN
acc
hyd
η18 α16 β34 TT α17 η19 η20 β35 TT α18 TTT
900 910 920 930 940 950 960 970
P140_A ALTFTNKNNPQRNQLLLRSLLGTIPVLINKSGDSNDQFNKDSEQKWDKTETNEGNLPGFGEVNGLYNAALLHTYGFFGTNTNST
acc
hyd
β36 TT β37 (Va) TT β38 (Vb) TT β39 (Vc) TT TT β40 (Vd) α19 η21
980 990 1000 1010 1020 1030 1040 1050 1060
P140_A DPKIGFKADSSSSSSSTLVGSGLNWTSQDVGNLVVINDTSFGFQLGGWFITFTDFIRPRTGYLGITLSSLQDQTIIWADQPWTS
acc
hyd

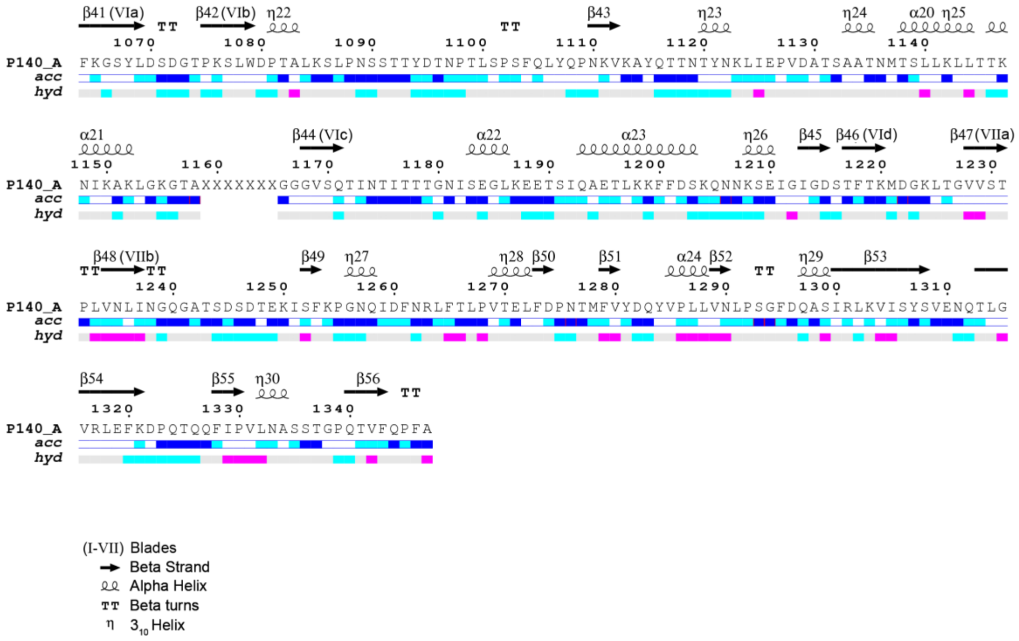

Supplementary Figure 3. **P140 sequence and secondary structural elements**

Amino acid sequence of the extracellular region of P140, with the corresponding secondary structural elements (helices with squiggles, strands with arrows and turns with TT letters) (Endscript2 [2]). The seven blades (or β-sheets) in the propeller are represented by roman numerals (I-VII). Bars below the sequence show solvent accessibility (blue is accessible, cyan is intermediate, white is buried) and hydropathy (pink is hydrophobic, cyan is hydrophilic).

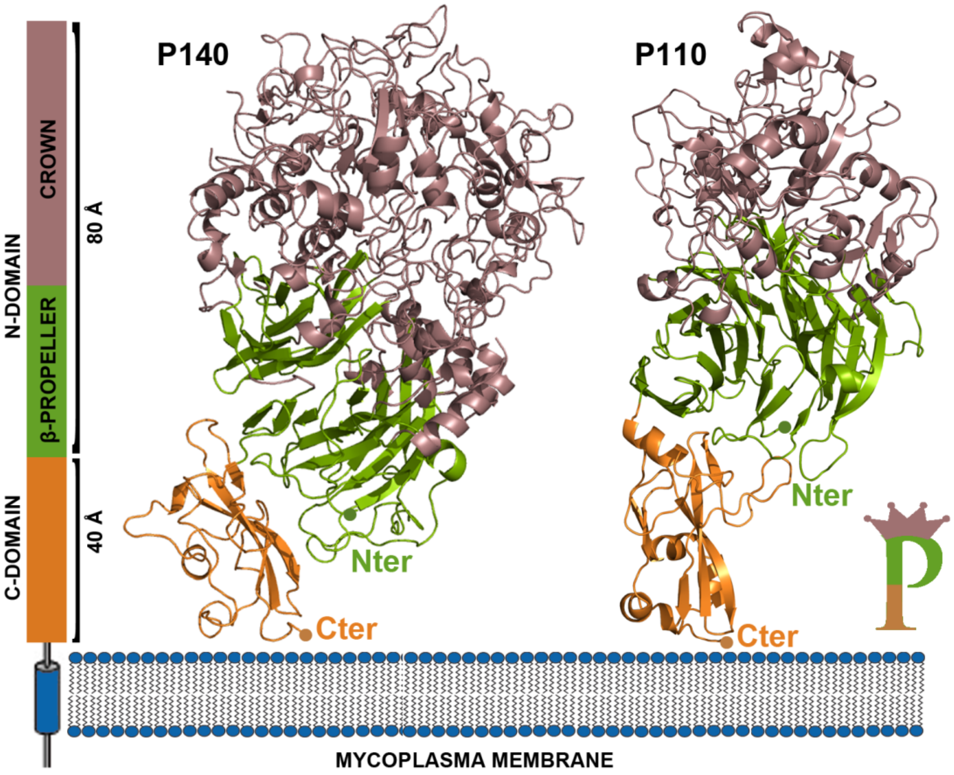

Supplementary Figure 4. **Structural comparison of P140 and P110**

P140 and P110 present a similar domain organization with small C-domains (orange) and large N-domains in the extracellular regions of the proteins. The N-terminal domains contain two distinct regions, a seven blade β-propeller (green) and a crown (brown), with the β-propellers situated between the C-terminal domains and the crowns. Also in both proteins, the C-terminal domains are followed in sequence by a (predicted) transmembrane helix and a cytosolic region with about a hundred residues.

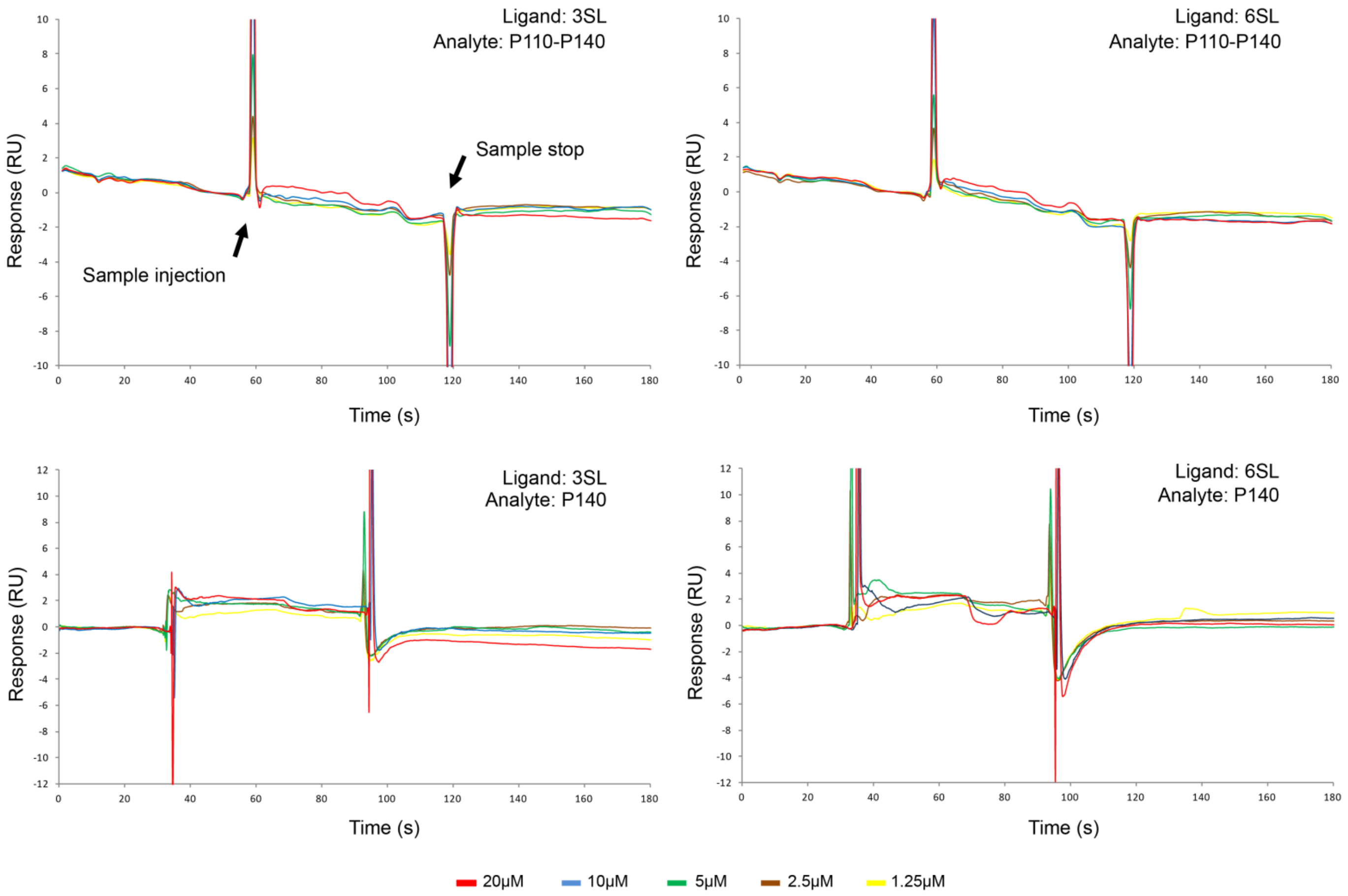

Supplementary Figure 5. Surface Plasmon Resonance (SPR)

Surface Plasmon Resonance sensorgrams showing no binding for the extracellular P140-P110 heterodimer (top panels) and for P140 (bottom panels) against increasing amounts of 3SL (left panels) and 6SL (right panels).

a

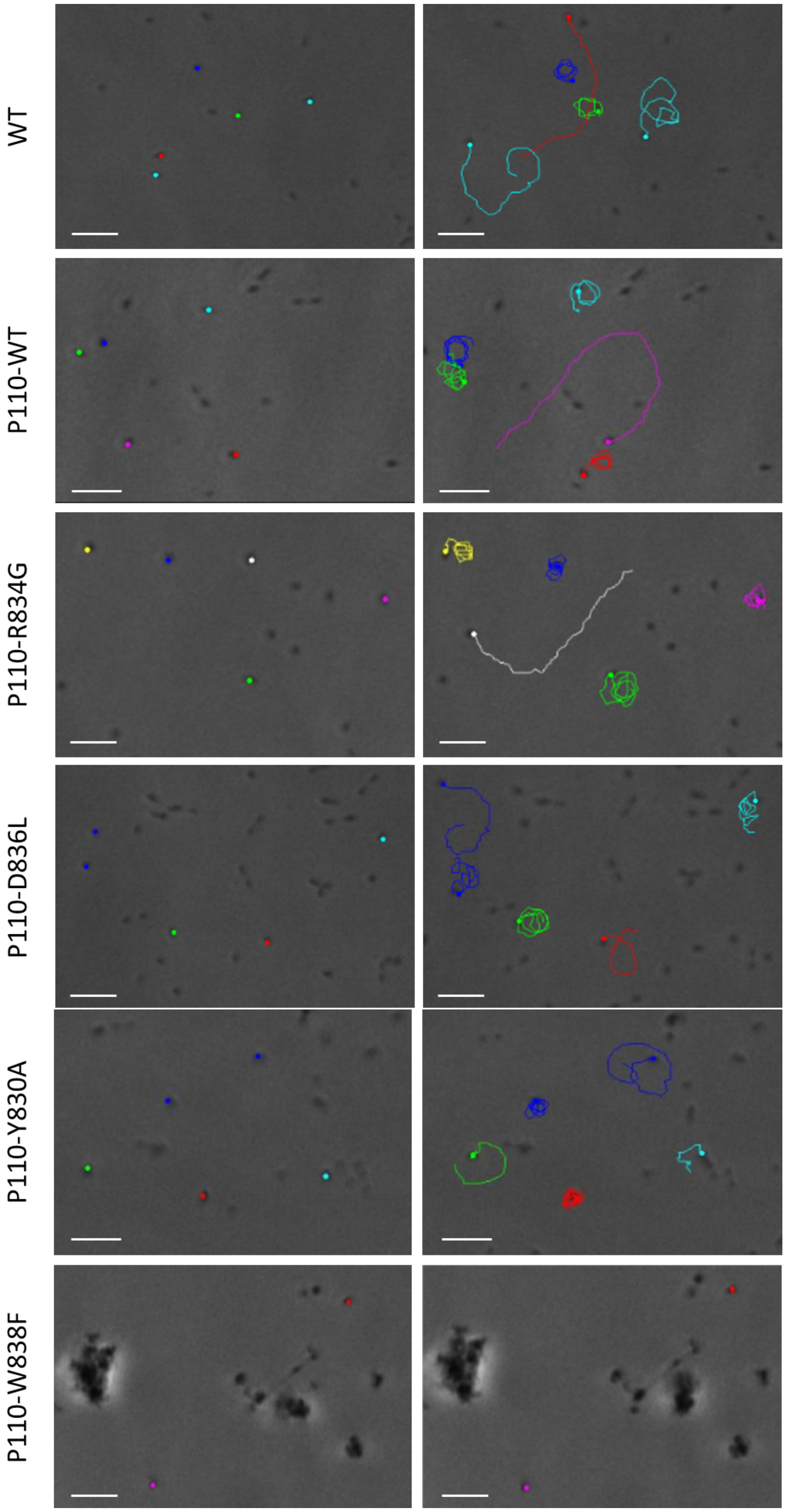

b

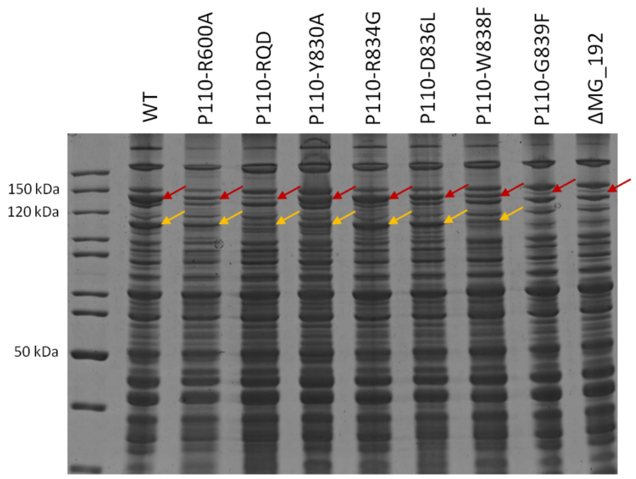

Supplementary Figure 6. Time-lapse microcinematography and expression analysis for WT and selected strain variants

a) Representative tracks of *M. genitalium* G37 Wild Type (WT) and P110-WT cells, and P110-R834G, P110-D836L, P110-Y830A and P110-W838F mutant cells. Although cells from P110-W838F were found to have severe hemadsorption deficiencies by flow cytometry, these cells attached properly to the plastic surface of Ibidi slides. This behaviour is somewhat common and has been previously described [3]. Bar is 5 µm. b) SDS-PAGE of whole cell lysates from the WT and the different adhesin mutants. Red and orange arrows indicate the presence of P140 and P110, respectively. Between two and four independent clones from each strain were analysed by SDS-PAGE and representative clones from each strain are shown here (original SDS-PAGE is provided independently).

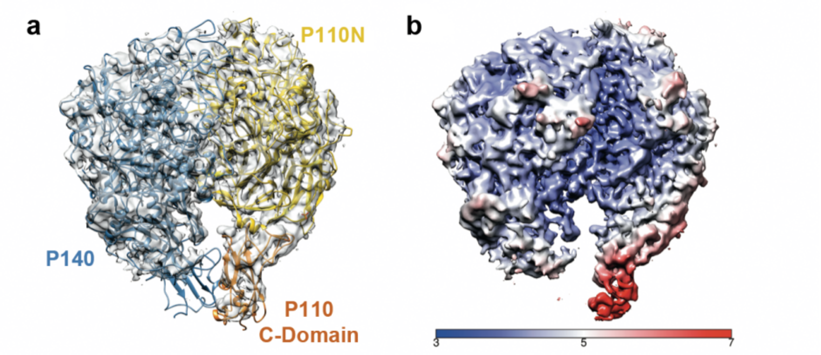

Supplementary Figure 7. **Cryo-EM of the P140-P110 extracellular region**

a) Cryo-EM map of the extracellular region of the P140-P110 heterodimer. The structure of P140-P110N, fitted into the map density, is also shown with P140 in blue and P110 in yellow. There is no density for the C-domain of P140, while density is visible, although weak, for the C-domain of P110 that was fitted independently into the cryo-EM map (orange). b) Local resolution of the cryo-EM map. The lowest resolution corresponds to the C-terminal domains, which indicates a significant flexibility of these domains, in particular of the C-terminal domain from P140, with respect to the bulkier N-domains.

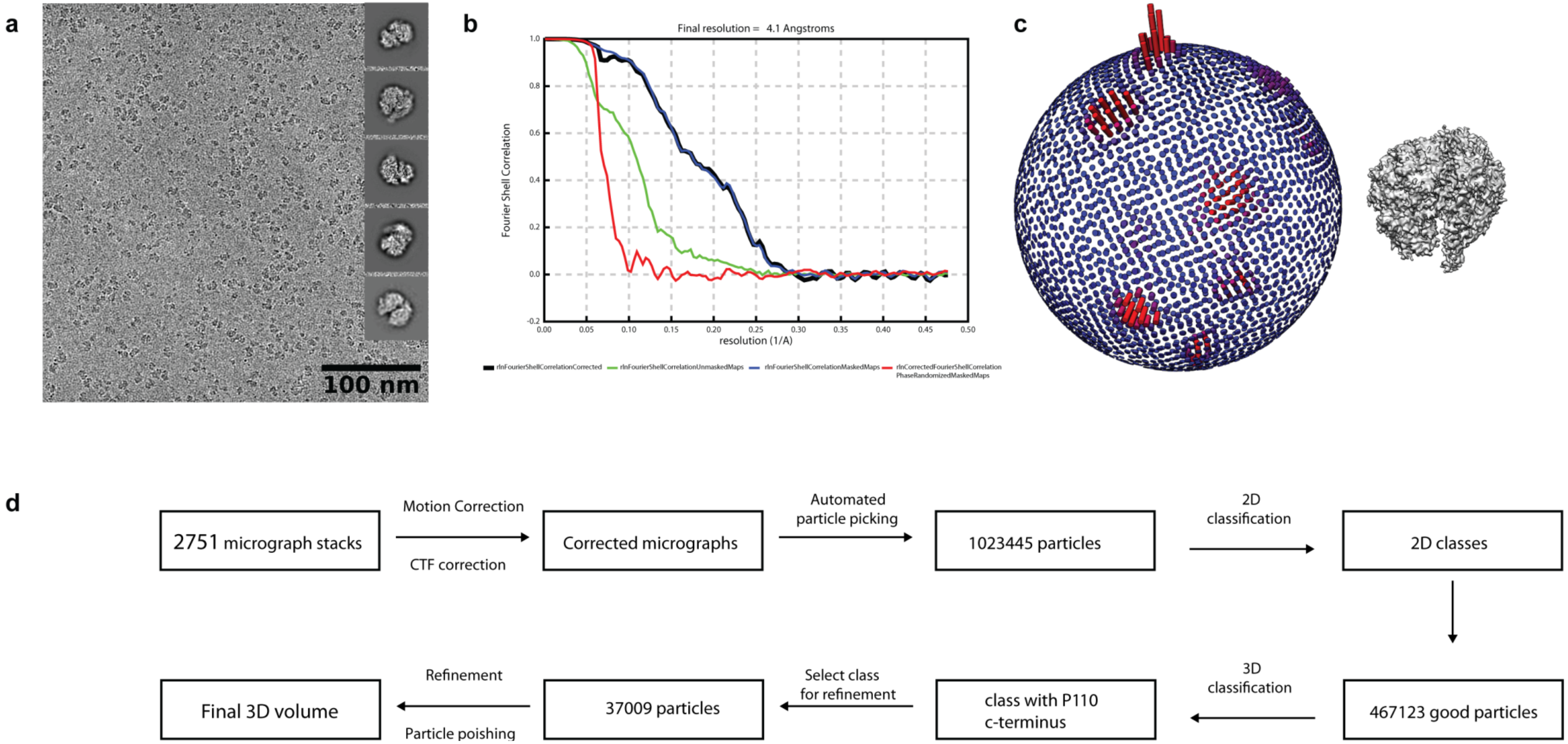

Supplementary Figure 8. **Processing of Cryo-EM data from the extracellular regions of P140-P110** a) Representative cryo-electron micrograph of P140-P110 heterodimers with a subset of 2D classes as insets (2751 micrographs were recorded in total). b) Fourier shell correlation showing a resolution of 4.1 Å using the 0.143 criteria. c) Angular distribution plot of images corresponding to the final 3D volume that displays anisotropy. d) Flowchart showing the processing pipeline from the micrograph stacks to the final 3D volume.

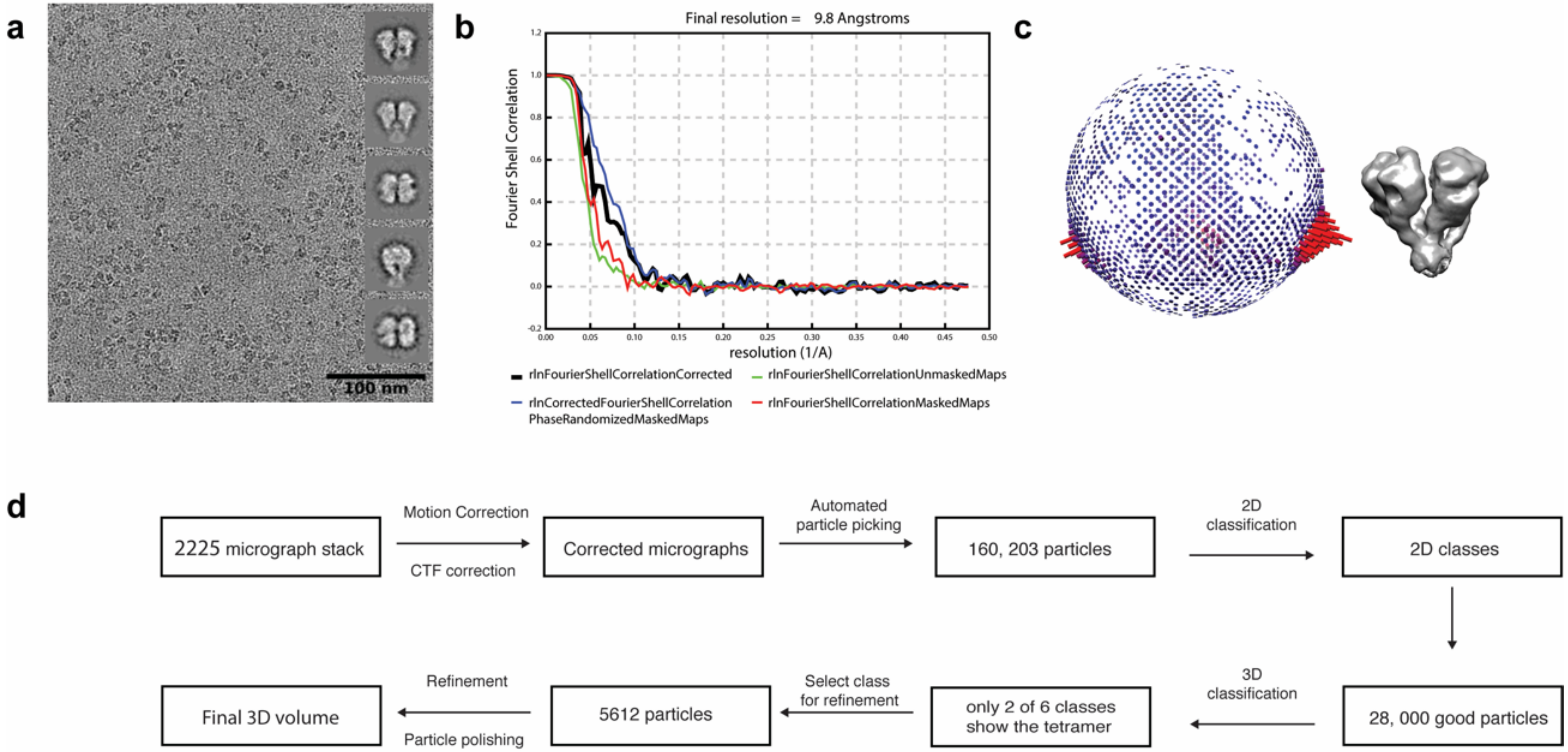

Supplementary Figure 9. **Image and data cryo-EM processing of the Nap complex** a) Representative cryo-electron micrograph of Nap complexes with a subset of 2D classes as insets (2225 micrographs were recorded in total). b) Fourier shell correlation of the cryo-EM map showing a resolution of 9.8 Å. c) Angular distribution plot of the final 3D volume. d) Flowchart showing the processing pipeline from the micrograph stacks to the final 3D volume.

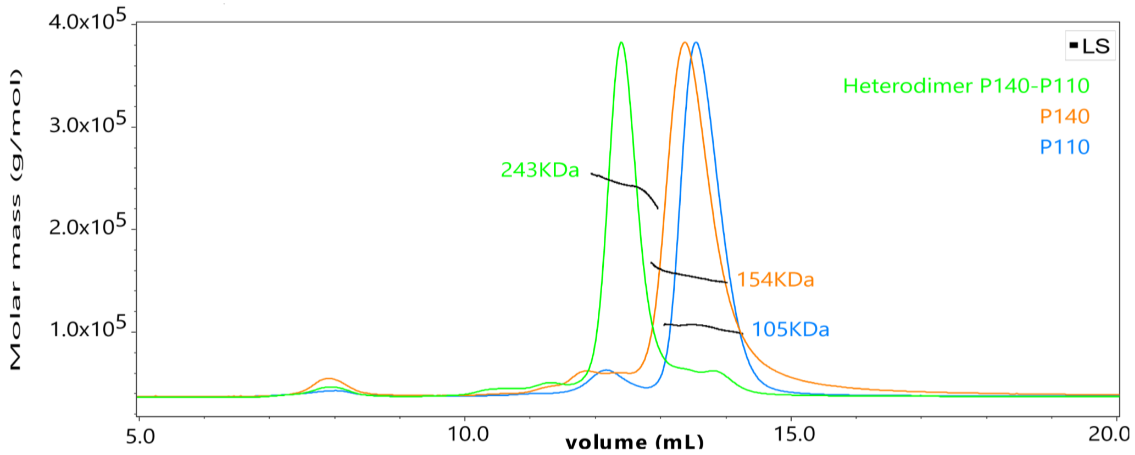

Supplementary Figure 10. SEC-MALS measurements

MALS measurements indicate that essentially only heterodimers are detected when mixing equimolecular amounts of P110 and P140.

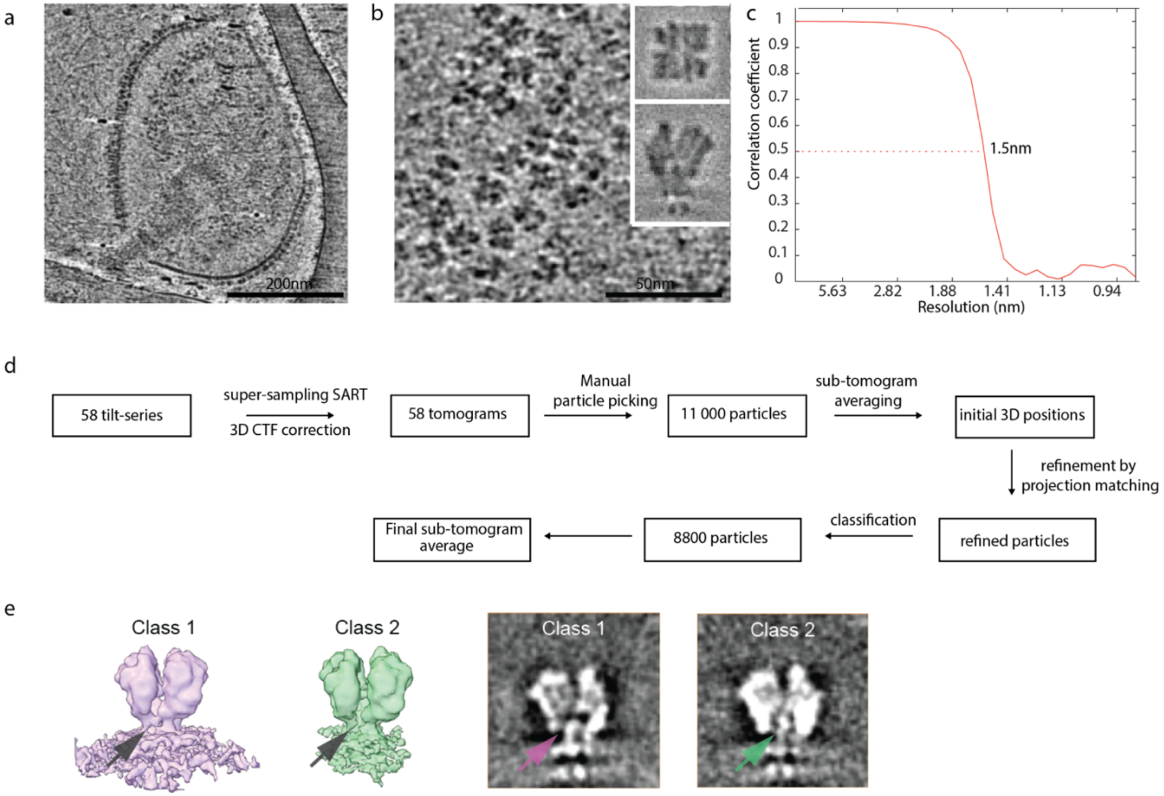

Supplementary Figure 11. **Cryo-ET of *M. genitalium* cells**. a) A 0.88 nm thick slice through a representative tomogram (58 tomograms were used in total) of a detergent-permeabilized *M. genitalium* cell. b) A zoomed in top view of the Nap complexes found on the cell in (a). Insets show slices through the top view (upper inset) and side view (lower-inset) of the sub-tomogram average. c) Fourier shell correlation of the sub-tomogram average showing a resolution of 15 Å at the 0.5 criterion. d) Flowchart showing the processing pipeline from the raw tilt-series to the final sub-tomogram average. e) Different classes observed during subtomogram averaging, class 1 (in purple) shows well resolved stalks (arrows), whereas class 2 (in green) shows less well-resolved stalks. Particles from class 1 were used in the final average.

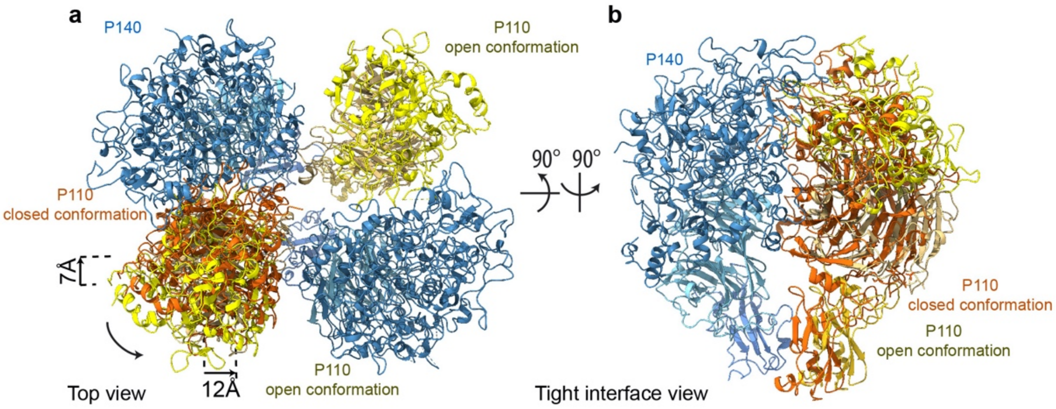

Supplementary Figure 12. Transition between the open and closed conformations through the movement of P110.

(a) Top view and (b) a tight interface view of a dimer of P140-P110 heterodimers showing the relative movement of P110 from the closed conformation (orange) as it retracts 7Å from its position at the tight interface and moves 12 Å closer to P140 across the loose interface to form the open conformation (yellow). P140 (blue) is kept unchanged as a reference.

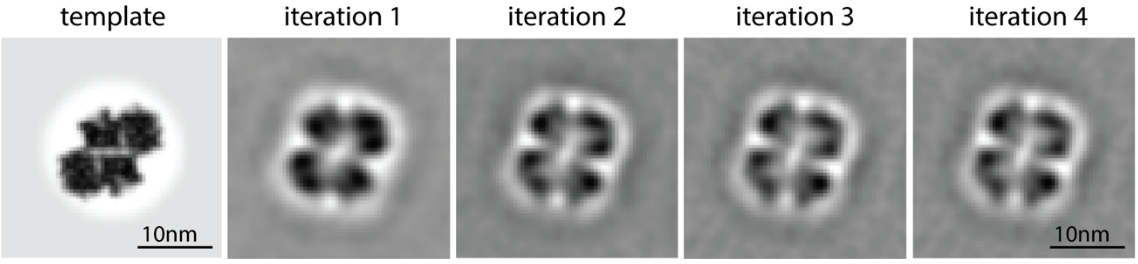

Supplementary Figure 13. Sub-tomogram averaging experiment using the crystal structure tetramer as a starting reference

When using the ‘closed' tetramer structure (crystal structure packing unit) as a starting reference, the sub-tomogram averaging procedure still converges to the open structure after 5 iterations. Scale bar is 10 nm.

Supplementary Tables

Data collection and Refinement Statistics [(a)]		
	**P140**	**P140-P110N**
Data collection		
**Space group**	C2	$P2_1$
**Cell dimensions**		
**a,b,c (Å)**	459.19, 116.65, 285.64	150.99, 157.28, 192.36
**α,β,γ (°)**	90.00, 124.20, 90.00	90.00, 93.81, 90.00
**Unique reflections**	123183 (6159)	189359 (784)
**Resolution (Å)**	189.88-3.26 (3.58-3.26)	157.28-2.65 (2.70-2.65)
**Wavelength (Å)**	0.9791	0.9789
**Rmeas (%)**[b]	0.15 (0.67)	0.58 (2.11)
**I/σI**	5.8 (1.6)	6.6 (1.5)
**CC(1/2)**	0.98 (0.74)	0.87 (0.343)
**Completeness (Spherical)**	63.5 (13.2)	73.3 (6.1)
**Completeness (Ellipsoidal)**	86.1 (57.9)	90.8 (44.4)
**Redundancy**	3.1 (2.6)	6.8 (6.8)
Refinement Statistics		
**Resolution**	37.36-3.26	121.66-2.65
**Num. of reflections**	123103	189358
**R_{Work} (%)**[c]	18.4	18.70
**R_{free} (%)**[d]	20.5	22.4
**No. residues**	1292	2134
**No. ligands**	0	0
**Solvent content (%)**	67.36	46
**Av. B-factor ($Å^2$)**	80.85	40.36
**Coor. Error (Å)**[e]	0.42	0.36
**Rms dev.bonds (Å)**	0.01	0.01
**Rms dev.angles (°)**	1.27	1.25

[a] **Values in parentheses correspond to the highest resolution shell.**

[b] **$R_{sym} = \Sigma_{hkl}\Sigma_i|I_i(hkl) - \langle I(hkl)\rangle| / \Sigma_{hkl}\Sigma_i I_i(hkl)$, where $I_i(hkl)$ is the intensity of an observation and $\langle I(hkl)\rangle$ is the mean value of observations for a unique reflection.**

[c] **$R_{cryst} = \Sigma_h|Fo(h) - Fc(h)| / \Sigma_h|Fo(h)$, where Fo and Fc are the observed and calculated structure-factor amplitudes, respectively.**

[d] **R_{free} was calculated with 5% of data, which was excluded from the refinement.**

[e] **Based on maximum likelihood.**

Supplementary Table 1. Data collection and refinement statistics

	P140-P110N	NAP complex
**Data collection**		
**Microscope**	FEI Titan Krios	FEI Titan Krios
**Detector**	Gatan K2 Summit	Gatan K2 Summit
**Acquisition software**	SerialEM 3.7	SerialEM 3.7
**Magnification**	130,000x	130,000x
**Pixel size (Å)**	1,05	1,05
**Total electron dose (e- / Å²)**	50	50
**Dose rate (Å2 / s-1)**	7,7	7,4
**number of frames**	32	34
**Defocus range (µm)**	-1 to -4	-1 to -4
**Micrographs used**	2.751	2.225
**Processing**		
**Software**	Relion 3.0 beta	Relion 3.0 beta
**Motion correction**	UCSF MotionCor2	UCSF MotionCor2
**CTF estimation**	GCTF v 1.06	GCTF v 1.06
**Total extracted particles**	1.024.402	160.203
**After 2D Classification**	467.123	28.000
**Number of 3D classes**	6	6
**Number of refined particles**	37.009	5.612
**Symmetry**	/	C2
**Map sharpening B factor (Å²)**	-78	-218
**Resolution FSC 0.143 (Å)**	4,1	9,8

Supplementary Table 2. Single particle analysis data collection

	Correlation#/clash-score^		
	Cryo-EM (P140-P110 heterodimer)	Cryo-EM (“Closed” Nap conformation)	Cryo-ET (“Open” Nap conformation)
**P140**	**0.74 / 13.55**	**-**	**-**
**P110**	**0.73 / 13.55**	**-**	**-**
**P140**	**-**	**0.95 / 12.13**	**0.94 / 10.39**
**P110**	**-**	**0.94 / 12.42**	**0.94 / 10.39**

Real-space correlation of the fitted models into the corresponding electron density maps. Fitting was based on the local optimisation algorithm of Chimera[4]
^The “clash-score” is defined as the number of serious overlaps (> 0.4 Å) per 1000 atoms (Molprobity [5]).

Supplementary Table 3. Correlation and clash-score values from rigid-body fitted crystal structures

Strain Name	Genotype	Reference
G37	Wild-type	ATCC 33530
ΔMG_192	Deletion of the MG_192 gene by allelic exchange (G37 ΔMG_192::*tet*M438)	Burgos et al. 2006
P110-WT	Re-introduction of a MG_192 wild-type allele in a ΔMG_192 mutant	Aparicio et al. 2018
P110-R600A	Introduction of a MG_192 allele bearing a R600A substitution in a ΔMG_192 mutant	This work
P110-RQD	Introduction of a MG_192 allele bearing a R600A, Q460A and D461A substitutions in a ΔMG_192 mutant	This work
P110-Y830A	Introduction of a MG_192 allele bearing a Y830A substitution in a ΔMG_192 mutant	This work
P110-R834G	Introduction of a MG_192 allele bearing a R834G substitution in a ΔMG_192 mutant	This work
P110-D836L	Introduction of a MG_192 allele bearing a D836L substitution in a ΔMG_192 mutant	This work
P110-W838F	Introduction of a MG_192 allele bearing a W838F substitution in a ΔMG_192 mutant	This work
P110-G389F	Introduction of a MG_192 allele bearing a G839F substitution in a ΔMG_192 mutant	This work

Supplementary Table 4. Strains used in this study

Strain	n	Motile cells (%)	Velocity (µm/s)[a]	Motility loss (%)
G37 (WT)	~250	88	0.126±0.002	-
P110-WT	~250	77	0.119±0.014	5.6
P110-Y830A	~250	78	0.100±0.011*	20.6
P110-R834G	~250	86	0.114±0.007	9.5
P110-D836L	~250	86	0.102±0.009*	19.0
P110-W838F	~64	0	0	100

[a]Velocities are shown as mean values +/- standard error. n=25 biologically independent cells, from different fields, different preparations and different microcinematographies.
* Statistically significant values ($T(25)=2.06$; $p<0.05$). Statistical significance was assessed with the Paired Student's two-sided T test.

Strain	Difference of means	Confidence interval (+/-)	Effect size	Degrees of freedom	p-value
WT	-	-	-	-	-
P110-WT	0.007	0.01663	0.16286	24	0.57
P110-Y830A	0.026	0.01522	0.69427	24	0.02
P110-R834G	0.012	0.00988	0.48446	24	0.10
P110-D836L	0.024	0.01230	0.81200	24	0.01
P110-W838F	-	-	-	-	-

Supplementary Table 5. Gliding motility parameters of *M. genitalium* G37 wild-type cells and all different mutant strains.

Synthetic clone for P140

CGAATTGAAGGAAGGCCGTCAAGGCCGCATTTAATTAAACAAGTTTGTACAAAAAAGCAGGCTCTGGAAGTTCTGTTTCAGGGTCCGAGCGGTGCAATGCATC
AGCCGAAAAAACGTCTGGCAAAAAAAAGCTGGGCATTTCTGACCGCAGCACTGACCCTGGGTGTTATTACCGGTGTTGGTGGTTATTTTCTGTTTAACCAGAAT
AAACAGCGTAGCAGCGTTAGCAATTTTGCATATCAGCCGAAACAGCTGAGCGTTAAACATCAGCAGGCAGTTGATGAAACCCTGACCCCGTGGACCTGGAATA
ATAATAATTTTTCAAGCCTGAAAATTACCGGTGAAAATCCGGGTAGCTTTGGTCTGGTTCGTAGCCAGAATGATAATCTGAATATTAGCAGCGTGACCAAAAAT
AGCAGCGACGATAATCTGAAATATCTGAATGCCGTCGAGAAATATCTGGATGGTCAGCAGAATTTTGCCATTCGTCGCTATGATAATAATGGTCGTGCCCTGTA
TGATATTAACCTGGCAAAAATGGAAAATCCGAGCACCGTTCAGCGTGGTCTGAATGGTGAACCGATTTTTGATCCGTTTAAAGGTTTTGGTCTGACCGGTAATG
CACCGACCGATTGGAATGAAATTAAAGGTAAAGTTCCGGTTGAGGTTGTTCAGAGTCCGCATAGCCCGAATCTGTATTTTGTTCTGCTGGTTCCGAAAGTTGCC
CTGGAATACCATAATCTGAACAATCAGGTGGTGAAAGAAAGCCTGGAAGTTAAAGCAACCCAGAGCAGCTTTAATCCGACCCAGCGTCTGCAGAAAGATAGT
CCGGTTAAAGATAGCAGCAAACAGGGTGAAAAACTGAGCGAAACCACCGCAAGCAGCATGAGCAGCGGTATGGCAACCAGCACCCGTGCAAAAGCACTGAA
AGTTGAAGTTGAACGTGGTAGCCAGAGCGATAGCCTGCTGAAAAATGATTTTGCAAAAAAACCGCTGAAGCATAAAAACAGCAGCGGTGAAGTTAAACTGGA
AGCCGAAAAAGAATTTACCGAAGCATGGAAACCGCTGCTGACCACCGATCAGATTGCACGTGAAAAAGGTATGGGTGCAACCGTTGTTAGCTTTTATGATGCA
CCGTATAGCGAAAATCATACCGCATTTGGTCTGGTGGATCATATTGATCCGAAAAAAATGGTCGAGAATTATCCGCCTAGCTGGAAAACCCCGAAATGGAATC
ATCATGGCATCTGGGATTATAATGCACGTAATCTGCTGCTGCAGACCACCGGTTTTTTTAATCCGCGTCGTCATCCGGAATGGTTTGATGAAGGTCAGGCAAAA
GCAGATAATACCAGTCCGGGTTTTAAAGTTGGTGATACCGATCATAAAAAAGATGGCTTTAAAAAAAAATAGCTCCAGCCCGATTGCACTGCCGTTTGAAGCAT
ATTTTGCCAATATTGGTAATATGGTGGCCATTGGTAACAGCGTGTTTATTTTTGGTGGTAATGGTCATGCCACCAAAATGTTTACCACCAATCCGCTGAGCATTG
GTGTGTTTCGTATCAAATATACCGATAATTTTAGCAAAAGCAGCGTTACCGGTTGGCCGTATGCAGTTCTGTTTGGTGGTCTGATTAATCCGCAAACCAATGGT
CTGAAAGATCTGCCGCTGGGCACCAATCGTTGGTTTGAATATGTTCCGCGTATGGCAGTTAGCGGTGTTAAATGGGTTGGTAATCAGCTGGTTCTGGCAGGCAC
CCTGACCATGGGTGATACCGCAACCGTTCCGCGTCTGAAATATGATCAGCTGGAAAAACATCTGAATCTGGTTGCACAGGGTCAGGGTCTGCTGCGTGAAGAT
CTGCAGATTTTTACCCCGTATGGTTGGGCAAATCGTCCGGATATTCCGGTTGGTGCATGGCTGCAGGATGAAATGGGTAGCAAATTTGGTCCGCACTATTTTCT
GAACAACCCGGATATTCAGGACAATGTGAATAATGATACCGTGGAAGCACTGATCAGCAGCTATAAAAATACCGACAAACTGAAACACGTGTATCCGTATCGT
TATAGCGGTCTGTATGCATGGCAGCTGTTTAATTGGAGCAATAAACTGACCAATACACCGCTGAGCGCCAATTTTGTTAACGAAAATAGCTATGCACCGAATA
GCCTGTTTGCAGCAATTCTGAATGAAGATCTGCTGACCGGTCTGAGCGATAAAATTTTTTATGGCAAAGAGAACGAATTTGCCGAAAATGAAGCCGATCGTTTT
AATCAGCTGCTGAGCCTGAATCCGAATCCGAATACCAATTGGGCACGTTATCTGAATGTTGTTCAGCGTTTTACCACCGGTCCGAATCTGGATAGCAGCACCTT
TGATCAGTTTCTGGATTTTCTGCCGTGGATTGGTAATGGTAAACCGTTTAGCAATAGCCCGAGCCCGAGCACCAGCGCCAGCAGTAGTACACCGCTGCCGACCT
TTAGCAATATTAATGTTGGTGTGAAAAGCATGATCACCCAGCATCTGAATAAAGAAAATACCCGCTGGGTGTTTATCCCGAATTTTAGTCCGGATATTTGGACC
GGTGCAGGTTATCGTGTTCAGAGCGCAAATCAGAAAAATGGTATTCCGTTCGAACAGGTTAAACCGAGCAATAATAGCACCCCGTTTGATCCGAATAGCGACG
ATAACAAAGTTACCCCGAGCGGTGGTAGCAGCAAACCGACCACCTATCCGGCACTGCCGAATAGCATTAGCCCGACCAGCGATTGGATTAATGCACTGACCTT
TACCAACAAAAATAATCCGCAGCGTAACCAACTGCTGCTGCGTAGCCTGCTGGGCACCATTCCGGTTCTGATTAATAAAAGCGGTGATAGCAACGATCAGTTC
AACAAAGATAGCGAACAGAAATGGGACAAAACCGAAACCAATGAAGGTAATCTGCCTGGTTTTGGTGAAGTTAATGGTCTGTATAATGCAGCTCTGCTGCACA
CCTATGGTTTTTTTGGCACCAATACCAATAGCACCGATCCGAAAATTGGCTTTAAAGCAGATAGCAGCAGCTCAAGTAGCAGCACCCTGGTTGGTAGCGGTCTG
AATTGGACCAGCCAGGATGTTGGTAATCTGGTTGTTATTAACGATACCAGCTTTGGTTTTCAGCTGGGTGGTTGGTTTATTACCTTTACCGATTTCATTCGTCCG
CGTACCGGTTATCTGGGTATTACCCTGAGCAGCCTGCAGGATCAGACCATTATTTGGGCAGATCAGCCGTGGACCAGCTTTAAAGGTAGCTATCTGGATAGTGA
TGGCACCCCGAAAAGCCTGTGGGATCCGACCGCACTGAAAAGCCTGCCGAATAGTAGCACCACCTATGATACCAATCCGACCCTGAGCCCGAGCTTTCAGCTG
TATCAGCCGAATAAAGTTAAAGCATATCAGACCACCAATACCTATAATAAACTGATTGAACCGGTTGATGCAACCAGCGCAGCAACCAATATGACCAGTCTGC
TGAAACTGCTGACCACGAAAAACATTAAAGCCAAACTGGGTAAAGGCACCGCCAGCAGCCAGGGTAATAATAATGGTGGTGGTGTTAGCCAGACCATTAATA
CCATTACCACCACCGGTAATATTAGCGAAGGTCTGAAAGAAGAAACCAGCATTCAGGCAGAAACCCTGAAAAAATTCTTCGATAGCAAACAGAATAATAAAA
GCGAAATCGGCATTGGCGATAGCACCTTTACCAAAATGGATGGTAAACTGACCGGTGTTGTTAGCACACCGCTGGTTAATCTGATTAATGGTCAGGGTGCAAC
CAGCGATAGCGATACCGAAAAAATTAGCTTTAAACCGGGTAACCAGATCGACTTTAATCGTCTGTTTACCCTGCCGGTTACCGAACTGTTTGATCCTAATACCA
TGTTCGTGTATGATCAGTATGTTCCGCTGCTGGTGAATCTGCCGAGCGGTTTTGATCAGGCAAGCATTCGTCTGAAAGTGATTAGCTATAGCGTGGAAAATCAG
ACCCTGGGCGTTCGTCTGGAATTTAAAGATCCGCAGACCCAGCAGTTTATTCCGGTGCTGAATGCAAGCAGCACCGGTCCGCAGACCGTTTTTCAGCCGTTTAA
TCAGTGGGCAGATTATGTTCTGCCGCTGATTGTTACCGTTCCGATTGTTGTGATTATTCTGAGCGTTACCCTGGGTCTGACCATTGGTATTCCGATGCATCGTAA
TAAAAAAGCACTGCAGGCAGGTTTTGACCTGAGCAATAAAAAAGTTGACGTTCTGACCAAAGCCGTTGGTAGCGTTTTTAAAGAAATTATTAACCGCACCGGC
ATTAGCAATGCACCGAAAAAACTGAAACAGGCAACCCCGACCAAACCGACCCCGAAAACCCCTCCGAAACCGCCTGTTAAACATAAGTAAACCCAGCTTTCTT
GTACAAAGTGGTGGCGCGCCCTGGGCCTCATGGGCCTTCCTTTCACTGCC

Synthetic clone for P110

CGAATTGAAGGAAGGCCGTCAAGGCCGCATTTAATTAAACAAGTTTGTACAAAAAAGCAGGCTCTGGAAGTTCTGTTTCAGGGTCCGAGCGGTGCA
ATGAAAACCATGCGTAAACAAATTTATAAAAAAGCCTATTGGCTGCTGCTGCCGTTTCTGCCGCTGGCACTGGCAAATACCTTTCTGGTTAAAGAGGATAGCA
AAAACGTTACCGCATATACCCCGTTTGCAACCCCGATTACCGATAGCAAAAGCGATCTGGTTAGCCTGGCACAGCTGGATAGCAGCTATCAGATTGCAGATCA
GACCATTCATAACACCAACCTGTTTGTGCTGTTCAAAAGCCGTGATGTGAAAGTGAAATATGAAAGCAGCGGCAGCAACAACATTAGCTTTGATAGCACCAGC
CAGGGTGAAAAACCGAGCTATGTTGTTGAATTTACCAACAGCACCAATATCGGCATTAAATGGACCATGGTGAAGAAATATCAGCTGGATGTTCCGAATGTGA
GCAGCGATATGAATCAGGTTCTGAAAAACCTGATTCTGGAACAGCCGCTGACCAAATATACCCTGAATAGCAGCCTGGCAAAAGAAAAAGGTAAAACCCAGC
GTGAAGTTCATCTGGGTAGCGGTCAGGCAAATCAGTGGACCAGCCAGCGTAATCAGCATGATCTGAATAATAATCCGAGCCCGAATGCAAGCACCGGTTTTAA
ACTGACCACCGGTAATGCATATCGTAAACTGAGCGAAAGCTGGCCGATTTATGAACCGATTGATGGCACCAAACAGGGTAAAGGTAAAGATAGCAGCGGTTG
GAGCAGCACCGAAGAAAATGAAGCAAAAAATGATGCACCGAGCGTTAGCGGTGGTGGTAGCAGCAGCGGCACCTTTAATAAATATCTGAATACCAAACAGGC
CCTGGAAAGCATTGGTATTCTGTTTGATGATCAGACACCGCGTAATGTTATTACCCAGCTGTATTATGCAAGCACCAGCAAACTGGCAGTGACCAATAATCATA
TTGTGGTTATGGGCAATAGCTTTCTGCCGAGCATGTGGTATTGGGTTGTTGAACGTAGCGCACAAGAAAATGCCAGCAATAAACCGACCTGGTTTGCAAATAC
CAATCTGGATTGGGGTGAGGATAAACAGAAACAGTTTGTTGAAAACCAGCTGGGCTATAAAGAAACCACCAGTACCAATAGCCACAACTTTCATAGCAAAAG
CTTTACCCAGCCTGCATATCTGATTAGCGGTATTGATAGCGTGAACGATCAGATTATCTTCAGCGGTTTTAAAGCAGGTAGCGTTGGTTATGATAGCAGCAGCA
GCAGTAGCAGCTCAAGCAGCAGCTCAACCAAAGATCAGGCACTGGCATGGTCAACCACCACCAGTCTGGATAGCAAAACCGGTTATAAAGATCTGGTGACCA
ATGATACCGGTCTGAATGGTCCGATTAATGGTAGCTTTAGCATCCAGGATACCTTTAGCTTTGTTGTTCCGTATAGCGGCAATCATACCAATAATGGCACCACC
GGTCCGATTAAAACCGCATATCCGGTTAAAAAAGATCAGAAAAGCACCGTGAAAATTAATAGCCTGATTAATGCCACACCGCTGAATAGCTATGGTGATGAAG
GTATTGGTGTTTTTGATGCACTGGGCCTGAACTATAACTTCAAAAGCAATCAAGAACGTCTGCCGAGCCGTACCGATCAGATTTTTGTTTATGGTATTGTGAGC
CCGAATGAACTGCGTAGCGCAAAAAGCAGCGCAGATAGCACCGGTAGCGATACCAAAGTTAATTGGAGCAATACCCAGAGCCGTTATCTGCCGGTTCCGTATA
ATTATAGCGAAGGTATTATTGATGCCGACGGTTTTAAACGTCCGGAAAATCGTGGTGCAAGCGTTACCACCTTTAGCGGTCTGAAAAGCATTGCACCGGATGG
TTTTGCAAATAGCATTGCAAATTTTAGCGTGGGTCTGAAAGCAGGTATTGATCCGAATCCGGTTATGAGCGGTAAAAAAGCAAATTATGGTGCCGTTGTTCTGA
CCCGTGGTGGTGTTGTTCGTCTGAATTTTAATCCGGGTAATGATAGCCTGCTGAGCACCACCGATAATAATATTGCACCGATCAGCTTTAGCTTTACCCCGTTTA
CCGCAGCAGAAAGCGCAGTTGATCTGACCACCTTTAAAGAAGTGACCTATAACCAAGAAAGCGGTCTGTGGTCCTATATTTTTGATAGCAGCCTGAAACCGAG
CCATGATGGTAAACAGACACCGGTTACCGACAATATGGGTTTTAGCGTTATTACCGTTAGCCGTACCGGTATTGAACTGAATCAGGATCAGGCAACCACCACC
CTGGATGTTGCACCGAGCGCACTGGCAGTTCAGAGCGGTATTCAGAGCACCACCCAGACCCTGACCGGAGTTCTGCCGCTGAGCGAAGAATTTAGCGCAGTTA
TTGCAAAAGATAGCGATCAGAATAAAATCGACATTTACAAAAATAATAATGGCCTGTTTGAAATTGATACCCAGCTGAGCAATAGCGTTGCAACCAATAATGG
TGGTCTGGCACCGAGCTATACCGAAAATCGTGTTGATGCATGGGGCAAAGTTGAATTTGCAGATAATTCAGTTCTGCAGGCACGTAATCTGGTTGATAAAACC
GTGGATGAGATTATTAACACACCGGAAATCCTGAACAGCTTTTTTCGTTTTACCCCTGCATTCGAAGATCAGAAAGCAACCCTGGTGGCAACCAAACAGAGCG
ATACCAGCCTGAGCGTTAGTCCGCGTATTCAGTTTCTGGATGGCAATTTTTATGACCTGAATAGCACCATTGCCGGTGTTCCGCTGAATATTGGTTTTCCGAGCC
GTGTTTTTGCAGGTTTTGCAGCACTGCCTGCATGGGTTATTCCGGTTAGCGTTGGTAGCAGCGTTGGCATTCTGTTTATTCTGCTGGTTCTGGGTCTGGGTATTG
GTATTCCGATGTATCGTGTTCGTAAACTGCAGGATGCCAGCTTTGTGAACGTGTTTAAAAAAGTTGATACCCTGACCACCGCAGTTGGTAGCGTTTATAAAAAG
ATTATCACCCAGACCGGTGTGGTTAAAAAAGCACCGAGTGCACTGAAAGCAGCAAATCCGAGCGTTAAAAAACCGGCAGCATTTCTGAAACCGCCTGTTCAGC
CTCCGAGCAAACCGGAAGGTGAACAGAAAGCAGTTGAAGTTAAAAGCGAGGAAACCAAAAGCTAAACCCAGCTTTCTTGTACAAAGTGGTGGCGCGCCCTGG
GCCTCATGGGCCTTCCTTTCACTGCC

Supplementary Table 6. Synthetic clones obtained from Geneart. Start and stop codons of the full-length MG191 and MG192 genes are highlighted.

Primer name	Sequence
COMmg192-F	AGTGGGCCCACTAACAAAAAACAAATTAGTGATGTTGTTAGTGATTGTGTGAAAAAAATTGTTTATAATTAAGTTTGTATGAAAACAATGAGAAAACAG
COMmg192-R	AGTCTCGAGCTAACTTTTGGTTTCTTCTG
R600Amg192-F	CCTGAAAACGCGGGTGCTAGT
R600Amg192-R	ACTAGCACCCGCGTTTTCAGG
Q460AD461Amg192-F	AGTTTTTCAATCGCTGCCACCTTCAGCTTTG
Q460AD461Amg192-R	AAAGCTGAAGGTGGCAGCGATTGAAAAACTCCC
Y830Amg192-F	GGTTAGCACCTAGTGCCACAGAAAACAGGG
Y830Amg192-R	CCCTGTTTTCTGTGGCACTAGGTGCTAACC
R834Gmg192-F	GTTACACAGAAAACGGGGTTGATGCATGGG
R834Gmg192-R	CCCATGCATCAACCCCGTTTTCTGTGTAAC
D836Lmg192-F	CAGAAAACAGGGTTTTGGCATGGGGTAAAG
D836Lmg192-R	CTTTACCCCATGCCAAAACCCTGTTTTCTG
W838Fmg192-F	GTTGATGCATTTGGTAAAGTTG
W838Fmg192-R	CAACTTTACCAAATGCATCAAC
G839Fmg192-F	GATGCATGGTTTAAAGTTGAG
G839Fmg192-R	CTCAACTTTAAACCATGCATC
PacUp	GTAGCTAATCTAACAGTAGG
PacDw	GTCCTAGAACTTGGTGTATG
RTPCRmg192-F	TCCCCTAATGAATTGCGAAG
RTPRCmg192-R	CAGGGGCAATTGATTTAAGC
Tnp3	GATTCATGATTATATCGATCAAC
P110-F	AGGAGATATACCATGGCACTGGCAAATACCTTTC
P110-R	GTGATGGTGATGTTTAGGCAGTGCTGCAAAC
P110N-F	AGGAGATATACCATGGCACTGGCAAATACCTTTC
P110N-R	GTGATGGTGATGTTTTGCCAGACCACCATTATTGG
P140-F	AGGAGATATACCATGGGTGTTATTACCGGTGTTG
P140-R	GTGATGGTGATGTTTATCTGCCCACTGATTAAACG

Supplementary Table 7. Primers used in this study

References

1. Jones, D.T. Protein secondary structure prediction based on position-specific scoring matrices. *J Mol Biol* **292**, 195-202 (1999).
2. Robert, X. & Gouet, P. Deciphering key features in protein structures with the new ENDscript server. *Nucleic acids research* **42**, W320-W324 (2014).
3. Aparicio, D. et al. Mycoplasma genitalium adhesin P110 binds sialic-acid human receptors. *Nature Communications* **9**, 4471 (2018).
4. Pettersen, E.F. et al. UCSF Chimera--a visualization system for exploratory research and analysis. *J Comput Chem* **25**, 1605-12 (2004).
5. Davis, I.W., Murray, L.W., Richardson, J.S. & Richardson, D.C. MOLPROBITY: structure validation and all-atom contact analysis for nucleic acids and their complexes. *Nucleic Acids Res* **32**, W615-9 (2004).
