# Supplementary material for: Structure and mechanism of the Nap adhesion complex from the human pathogen Mycoplasma genitalium
Source: Nat Commun. 2020 Jun 8;11:2877. doi: 10.1038/s41467-020-16511-2 (PMC7280502; doi:10.1038/s41467-020-16511-2)
Supplement: Supplementary file 2 — Description of Additional Supplementary Files [file 41467_2020_16511_MOESM2_ESM.pdf]

Description of Additional Supplementary Files

File name: Supplementary Movie 1
Description: Rigid body fitting of P110 (in yellow) and P140N (in blue) into the cryo-EM density map (in gray), where the C-terminal domain of P140 was not resolved (Supplementary Figure 7). Towards the end of the movie, superposition with the crystal structure of the P140-P110N complex (purple, where P110N includes only the N-terminal domain) is shown. Despite the tight interactions between P140 and P110 there are no steric clashes between the modeled C-domains of the two subunits.

File name: Supplementary Movie 2
Description: The surface of the cryo-EM map of the Nap in beige with the fitted structures of P140 and P110, in blue and yellow respectively.

File name: Supplementary Movie 3
Description: The surface of the cryo-ET map of the Nap (in gray) showing the open conformation. The fitted structures of P140 and P110 are shown in blue and yellow respectively. In the first heterodimer the open conformation is fitted with an excellent fit, while in the second heterodimer the closed conformation is fitted leaving significant void space in the surface. Towards the end of the movie an additional P110 structure (in dark orange) is placed, where the difference in fit can be appreciated.

File name: Supplementary Movie 4
Description: The movie shows the transition of the P110 and P140 within the Nap from an open to the closed conformation with the animation of the subsequent release of the sialic acid receptor (in orange).
