# Supplementary material for: Structure and mechanism of the Nap adhesion complex from the human pathogen Mycoplasma genitalium
Source: Nat Commun. 2020 Jun 8;11:2877. doi: 10.1038/s41467-020-16511-2 (PMC7280502; doi:10.1038/s41467-020-16511-2)
Supplement: Supplementary file 8 — Reporting Summary [file 41467_2020_16511_MOESM8_ESM.pdf]

nature research

Corresponding author(s): Achilleas Frangakis
Last updated by author(s): 20-4-2020

Reporting Summary

Nature Research wishes to improve the reproducibility of the work that we publish. This form provides structure for consistency and transparency in reporting. For further information on Nature Research policies, see Authors & Referees and the Editorial Policy Checklist .

Statistics

For all statistical analyses, confirm that the following items are present in the figure legend, table legend, main text, or Methods section.

n/a	Confirmed	
☐	☒	The exact sample size (*n*) for each experimental group/condition, given as a discrete number and unit of measurement
☐	☒	A statement on whether measurements were taken from distinct samples or whether the same sample was measured repeatedly
☐	☒	The statistical test(s) used AND whether they are one- or two-sided<br>*Only common tests should be described solely by name; describe more complex techniques in the Methods section.*
☒	☐	A description of all covariates tested
☒	☐	A description of any assumptions or corrections, such as tests of normality and adjustment for multiple comparisons
☐	☒	A full description of the statistical parameters including central tendency (e.g. means) or other basic estimates (e.g. regression coefficient) AND variation (e.g. standard deviation) or associated estimates of uncertainty (e.g. confidence intervals)
☐	☒	For null hypothesis testing, the test statistic (e.g. *F*, *t*, *r*) with confidence intervals, effect sizes, degrees of freedom and *P* value noted<br>*Give P values as exact values whenever suitable.*
☒	☐	For Bayesian analysis, information on the choice of priors and Markov chain Monte Carlo settings
☒	☐	For hierarchical and complex designs, identification of the appropriate level for tests and full reporting of outcomes
☒	☐	Estimates of effect sizes (e.g. Cohen's *d*, Pearson's *r*), indicating how they were calculated

Our web collection on statistics for biologists contains articles on many of the points above.

Software and code

Policy information about availability of computer code

Data collection	The code is referenced. Freeware (SerialEM 3.7) and commercial code (DigitalMicrograph) has been used. All scripts are freely available.
Data analysis	The code is referenced.<br>Freeware (Coot 0.8, Xia2 0.3.6, Pymol 1.8.6, SerialEM, Relion 3.0 beta, Chimera & ChimeraX 0.88, MotionCorr2-01-30-2017, gctf v1.06, Artiatomi http://github.com/uermel/Artiatomi, https://github.com/lsprankel/Aparicio_2020) and commercial code (MATLAB 2019b) has been used. All scripts are freely available.

For manuscripts utilizing custom algorithms or software that are central to the research but not yet described in published literature, software must be made available to editors/reviewers. We strongly encourage code deposition in a community repository (e.g. GitHub). See the Nature Research guidelines for submitting code & software for further information.

Data

Policy information about availability of data

All manuscripts must include a data availability statement. This statement should provide the following information, where applicable:
- Accession codes, unique identifiers, or web links for publicly available datasets
- A list of figures that have associated raw data
- A description of any restrictions on data availability

Atomic coordinates and structure factors for the reported crystal structures from P140 and from the complex P140-P110N have been deposited into the Protein Data Bank under accession codes 6RUT and 6S3U, respectively. Cryo-electron microscopy densities were deposited in the EM Databank under the accession codes EMD-10261, EMD-10260 and EMD-10259 for the heterodimer, the single-particle cryo-EM Nap at in situ Nap from cryo-ET, respectively.

Field-specific reporting

Please select the one below that is the best fit for your research. If you are not sure, read the appropriate sections before making your selection.

- [x] Life sciences
- [ ] Behavioural & social sciences
- [ ] Ecological, evolutionary & environmental sciences

For a reference copy of the document with all sections, see nature.com/documents/nr-reporting-summary-flat.pdf

Life sciences study design

All studies must disclose on these points even when the disclosure is negative.

Sample size	cryo-EM: Sample size was chosen to such an amount in order to reach ~4A resolution; unsupervised classification was used to reject outliers;<br>cryo-ET: All Naps were selected; classification was used to cluster the data set;<br>Microcinematography: Sufficient cells were selected for a statistical significant p value;
Data exclusions	EM-studies: False positive particle selections were excluded through classification;<br>Microcinematography: No data was excluded. For motility 250 cells were analyzed. For velocities 25 cells were analyzed.
Replication	cryo-EM: Refinements and averages were done with random individual half sets.<br>All experimental findings could be replicated. For motility experiments, the frequency of motile cells was determined by examining approximately 250 isolated cells of each strain (Each cell is considered as a biological independent replicate). For the P110-W838F mutant, 64 individual cells were analyzed.
Randomization	2D and 3D classification for single particle analysis was performed in Relion. For tomography, principal components analysis was used to classify the particles and Relion - the results were similar.
Blinding	Investigators were not blinded during grouping since it is computationally performed

Reporting for specific materials, systems and methods

We require information from authors about some types of materials, experimental systems and methods used in many studies. Here, indicate whether each material, system or method listed is relevant to your study. If you are not sure if a list item applies to your research, read the appropriate section before selecting a response.

Materials & experimental systems

n/a	Involved in the study
x	Antibodies
x	Eukaryotic cell lines
x	Palaeontology
x	Animals and other organisms
x	Human research participants
x	Clinical data

Methods

n/a	Involved in the study
x	ChIP-seq
	x Flow cytometry
x	MRI-based neuroimaging

Flow Cytometry

Plots

Confirm that:

- [x] The axis labels state the marker and fluorochrome used (e.g. CD4-FITC).
- [x] The axis scales are clearly visible. Include numbers along axes only for bottom left plot of group (a 'group' is an analysis of identical markers).
- [x] All plots are contour plots with outliers or pseudocolor plots.
- [x] A numerical value for number of cells or percentage (with statistics) is provided.

Methodology

Sample preparation	Hemadsorption was quantified using flow cytometry as previously described. We used 109 mycoplasma cells during the hemadsorption assay.
Instrument	Fluorescence activated cell sorting (FACS) data were acquired using a FACSCalibur (Becton Dickinson, Franklin Lakes, USA) equipped with an air-cooled 488 nm argon laser and a 633 nm red diode laser

Software	CellQuest-Pro and FACSDiva software (Becton Dickinson)
Cell population abundance	Binding of mycoplasma cells to red blood cells were modeled in an inverse Langmuir isothermal kinetic function M_f=1-(B_max [RBC])/(K_d+ [RBC])
Gating strategy	To avoid the interfence of events related to SP4 medium, a double gating strategy was performed using a preliminary FL3-H/FL1-H gate following an SSC-H/FL1-H gate as thoroughly described in Garcia-Morales at al., 2014.

☒ Tick this box to confirm that a figure exemplifying the gating strategy is provided in the Supplementary Information.
